# Space Habitation and Microbiology: Status and Roadmap of Space Agencies

**DOI:** 10.1264/jsme2.ME2903rh

**Published:** 2014-09-01

**Authors:** Mark Ott, Duane Pierson, Masaki Shirakawa, Fumiaki Tanigaki, Masamitsu Hida, Takashi Yamazaki, Toru Shimazu, Noriaki Ishioka

**Affiliations:** 1 Human Health and Performance, Lyndon B. Johnson Space Center, National Aeronautics and Space Administration (NASA), 1601, NASA Parkway, Houston, Texas 77058, USA; 2 Space Environment Utilization Center, Human Spaceflight Mission Directorate, Japan Aerospace Exploration Agency (JAXA), 2–1–1 Sengen, Tsukuba, Ibaraki 305–8505, Japan; 3 Laboratory of Space and Environmental Medicine, Graduate School of Medicine, Teikyo University, 359, Ohtsuka, Hachiohji, Tokyo 192–0395, Japan; 4 Japan Space Forum, 3–2–1, Kandasurugadai, Chiyoda, Tokyo 101–0062, Japan; 5 Institute of Space and Astronautical Science, Japan Space Exploration Agency (JAXA), 3–1–1 Yoshinodai, Chuo-ku, Sagamihara, Kanagawa, 252–5210, Japan

## Microbiological research and operational efforts at the NASA

The ubiquitous nature of microbiology is reflected in the diversity of microbiological research and operational efforts at NASA. For example, the impact of microorganisms on other planets and the protection of Earth from the potential of microbial life elsewhere is the responsibility of the Office of Planetary Protection (http://planetaryprotection.nasa.gov/about). While the Office of Planetary Protection does not include forward or back contamination to or from a low Earth orbit, research platforms, such as the ISS, are being used to better understand the survival of microorganisms and corresponding contamination control in the extreme conditions of space (2, 4, 10). Another example is the NASA Center for Astrobiology, which focuses on the origin, evolution, distribution, and future of life in the universe (https://astrobiology.nasa.gov/). The large focus on microbiology is within its human exploration operations. NASA has historically set microbiological requirements, including stringent monitoring regimes, to mitigate risks to the health and performance of astronauts. Microorganisms can have both positive and negative impacts on many aspects of human spaceflight, including the risk and prevention of infectious diseases, performance of Environmental Control and Life Support Systems (ECLSS), spaceflight foods, and vehicle design and integrity. Even though a great amount of information has been obtained (1, 5, 7, 8), several key questions regarding the impact of microorganisms on human spaceflight still remain. Research into the uncertainties of risks that may affect crew health are the responsibility of the NASA Human Research Program (http://www.nasa.gov/exploration/humanresearch/). The research that addresses our fundamental understanding of space life science and its translation for benefits to the general public on Earth is the responsibility of NASA Space Biology.

Microbial research by the NASA Human Research Program has investigated changes in microbial hazards, infectious disease: dose-responses, and crew exposure that could impact the health and performance of the crew during a spaceflight mission. In order to achieve its goals, the NASA Human Research Program performs ground-based and spaceflight studies designed to decrease uncertainties in our knowledge of current risks entitled “Risk of Adverse Health Effects Due to Alterations in Host-Microorganism Interactions”. Details on the content of this research can be found at http://humanresearchroadmap.nasa.gov/Risks/?i=80. An example of studies being performed in this program is the spaceflight experiment, a study on the impact of long-term space travel on the astronaut’s microbiome, led by Principal Investigator Dr. Hernan Lorenzi of the J Craig Venter Institute. This study has been designed to evaluate changes in the astronaut microbiome that may be occurring during spaceflight missions, which could impact their health and performance. This experiment will use state-of-the-art techniques to determine fluctuations in the bacterial and viral microbiome of astronauts from key body sites before, during, and after a space mission. In addition, key immunological and environmental parameters will be monitored.

## Microbiology research in the NASA Space Biology Program

The NASA Space Biology Program performs basic microbiological research, including both ground-based and spaceflight studies, which enhances our understanding of microorganisms and their unique responses to spaceflight culture. The broad scope of microbiology research by the NASA Space Biology Program can be found in the Fundamental Space Biology Science Plan at http://www.nasa.gov/exploration/library/esmd_documents.html. An example of this research is the spaceflight experiment, an investigation of host-pathogen interactions, conserved cellular responses, and countermeasure efficacies during spaceflight using the human surrogate model *Caenorhabditis elegans*, led by Principal Investigator Dr. Cheryl Nickerson of the Biodesign Institute at Arizona State University. This study builds upon Dr. Nickerson’s previous findings that *Salmonella enterica* serovar Typhimurium displayed increases in virulence when cultured during spaceflight. In this latest experiment, changes in virulence and genetic regulation are being evaluated when both the host and pathogen are simultaneously exposed to the spaceflight environment. In addition to inflight monitoring of the infection process, this study will also evaluate the impact of altering media components to better define the mechanisms underlying this spaceflight effect.

The need for microbiology in NASA’s research goals was reinforced by the National Academy of Sciences in their 2011 report, “Recapturing a Future for Space Exploration: Life and Physical Sciences Research for a New Era” (6), commonly referred to as the Decadal Survey. Included in their recommendations was the need to:

1. Establish a “Microbial Observatory” program on the ISS,
2. Establish research analyzing microbial growth and physiological responses to the spaceflight environment, and
3. Develop research aimed at investigating microbial-plant systems in long-term life support systems.

These recommendations have established the groundwork for future microbiological research, which intersects with multiple NASA programs. NASA research will continue to provide exciting new findings and create opportunities for greater collaborations and utilization of the International Space Station (ISS) platform. Most importantly, the knowledge gained from studies, such as the “Microbial Observatory” initiative, hold the potential to improve the health and performance of spaceflight crews and translate into important findings for scientists on Earth.

## Current status of Japanese microbial study in space

The JAXA has solicited research opportunities onboard Kibo, and research related to microbial monitoring, as shown in Table 1, have been selected.

**Fig. 1:**
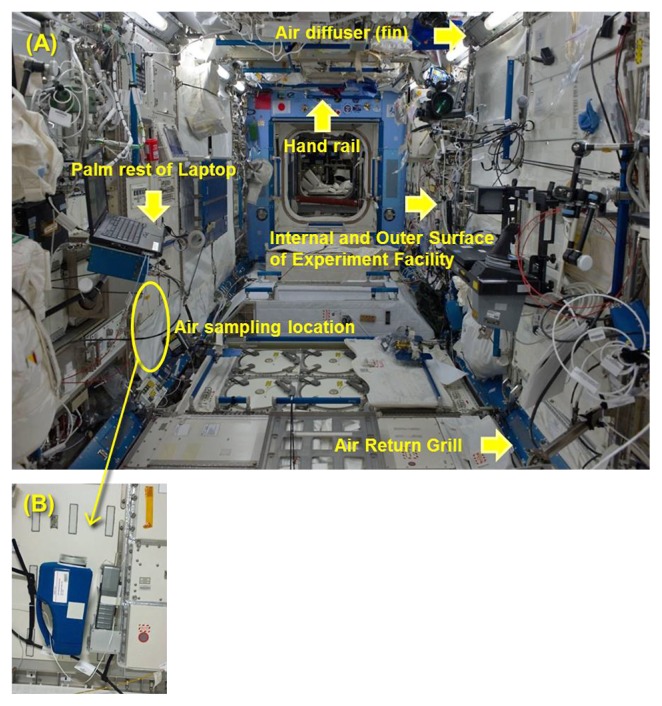
(A) Typical sampling location inside the Kibo module for “Microbe-1, 2, 3” experiments. (B) Air sampling with the NASA Air Sampler Device (left) and JAXA Particle Counter (right) in the Microbe-3 experiment.

“Microbe-1” to “Microbe-3” are a series of experiments that monitor microbial dynamics in the Kibo module and were initiated just before Kibo was launched. The objective of “Microbe” is to constantly monitor the microbes in Kibo that may affect crew members’ health and the ISS systems and facilities. Experiment data will be used to evaluate the stress caused by microbes on crew members as the medical impact of living on a space station. Fig. 1A shows a typical sampling location inside the Kibo module. The features of “Microbe” are as follows:

1. Adhesive sheets for microbial detection and collection, and swabs have been used.
2. In order to monitor microbes in the cabin air inside Kibo, an air sampling device and particle counter were used in “Microbe-3” (Fig. 1B).
3. Collected samples were retrieved and analyzed by molecular and biological analyses.

New hardware, a commercial handheld Particle Counter (KR-12A, Rion) that was customized for flight, was introduced in the “Microbe-3” experiment, (Fig. 1B [right]). This particle counter has the following functions: 1) Detect particles floating in the air; 2) Display a particle count for six ranges in size of ≥0.5 μm, ≥1.0 μm, ≥2.0 μm, ≥3.0 μm, ≥5.0 μm, and ≥10.0 μm; 3) Store up to 500 measurements; 4) Downlink the data through a laptop terminal in Kibo; 5) Operate for approximately 30 h with four D-cell alkaline batteries. The particle counter was launched by HTV-3 in July, 2012. During the “Microbe-3” experiment and used to count particles in the Kibo module in September-October timeframe, 2012.

Air samples were also collected at the same time using the NASA Air Sampling Device (ASD) for the SWAB project, which was borrowed from NASA (Fig. 1B [left]). JAXA fabricated a newly designed battery holder for the SWAB ASD.

On-going microbial monitoring in KIBO will be continued and expanded in the “Microbe-4” experiment. The objective of “Microbe-4” is to accumulate fundamental data on microbial dynamics in space habitation environments in order to define correct upper and lower thresholds for the indoor environmental quality control of air, water, and surfaces in this unique habitat.

Onboard microbial monitoring systems are planned for use in the “Microbe-A” experiment. These devices will not require sample recovery to the ground and will allow crew members to evaluate risks and impact promptly onboard Kibo. The purpose of “Microbe-A” is to develop and demonstrate a rapid and effective onboard monitoring system for crew members to monitor microorganisms in the ISS cabin environment. On-site and real-time microbial monitoring systems will be developed to protect crew members from harmful microorganisms. Crew members will be able to monitor microflora in their living environment on-orbit and immediately take countermeasures against microbial contamination. The development of onboard monitoring facilities based on ground-based microbial monitoring technologies has been studied. Candidates are Loop-Mediated Isothermal Amplification (called “LAMP”; http://loopamp.eiken.co.jp/e/tech/index.html) and a fluorescent microscope-based system for enumeration with a newly designed microfluidic device for cell counting (12).

## Future prospects of microbial research in ISS/Kibo

The JAXA president’s advisory committee to promote the utilization of ISS/Kibo developed “Kibo utilization scenarios (strategic plans) toward 2020” in March 2012. These scenarios were developed in the disciplines of life sciences, space medicine, and physical sciences (http://iss.jaxa.jp/en/kiboexp/scenario/). Future microbial research in the scenario for life sciences has been described as follows: “A priority item is ‘Research on the relationship between microbes and humans in space’. The ISS is a completely closed environment that offers a long-term microgravity environment. Therefore, it is a unique environment in which microbes can move and attach themselves to devices or humans (especially the exposed part of the body and head). Monitoring microbes inside Kibo and analyzing their movement must be continued to study their influence on humans and to prevent health hazards caused by these microbes during a long-term space mission. In addition, an integrated monitoring and analysis system needs to be established in cooperation with NASA (USA), ESA (EU), and ROSCOSMOS (Russia), not only for inside Kibo, but also the entire ISS.”

The scenario also states that the following area should be promoted:

1. Research on the relationship between humans and microbes should be promoted in the future as space microbiology, *e.g.* evaluation of the integral effects of microgravity or space radiation; propagation of cell genes as microbial ecosystems; space immunology research aimed at maintaining healthy life in space; opportunistic infections originating from the herpes virus or stress; and viruses related to fatigue
2. Probiotics, which actively use microbes in the space environment, will be a promising area in the future, *e.g.* metagenomic research of enteric bacterial flora from a lactic fermenting beverage or food intake is expected to contribute to the maintenance of astronaut health.
3. Biostimulation research on the application of producing food using excrement in space and the prevention of a depressed immune system will be necessary.
4. Examples of the application and improvement targets are relaxation during a long-term mission; air-cleaning technology; production capacity; measurement of photosynthetic ability using cyanobacteria; a comparison experiment of the absorption of carbon dioxide and the oxygen evolution effect on the ground; and development of fundamental technology for a new enclosed life support system.

## Future international cooperation of JAXA and other space agencies

The International Space Station program is one of the biggest international cooperative projects in the world. Onboard research should also be promoted by international collaborations. This collaboration will be necessary, especially for microbial research, because the cabin or internal environment inside the ISS is common and the environment will mutually affect each agency’s module, facilities, and onboard crew members. However, although microbial environment conditions are commonly assessed in terms of medical operations (*i.e.* a minimum screening level), microbial research to support or advance these activities (*e.g.* validation of the current threshold) have so far been conducted individually by each space agency. Therefore, JAXA has proposed the following possible international collaborations or activities to the other international partners.

1. Sharing of microbial observation data or samples is important for enabling various analyses by international research communities or agencies after the Primary Investigator’s specific analyses have been completed. Compiling data as a database and framework for exchanging information on a regular basis should also be considered.
2. Monitoring of other modules to expand the entire ISS is necessary for spatial comparisons (*e.g.* Japanese Kibo vs. Russian Service Module) of data. If data are exchanged, the sampling frequency rate in one module can increase.
3. The standardization of protocols or measures for monitoring is necessary to define unified upper and lower thresholds and the microbiological quality control of air, water, and surfaces. This activity may lead to the establishment of effective measures for ISS operations (*e.g.* reduce crew time for housekeeping by concentrated/prioritized cleaning). JAXA has proposed that JAXA or Japanese investigators can provide the sampling devices used in our experiments for this purpose.
4. The development of real-time and on-site monitoring systems is necessary to reduce returned samples. This system is also required to evaluate the microbial status onboard the ISS in a timely manner.

JAXA expects that microbial research on the ISS/Kibo is also beneficial on the ground such as the establishment of a process to monitor an environment that requires specific quality control, the spin-off of handy, reliable devices developed for space experiments, and applications to health and environmental control.

## Figures and Tables

**Table 1:** Microbial monitoring experiments selected by JAXA

Experiment Name	Experiment Title	Principle Investigator	Status	References
Microbe-1	Studies on microbiota on board	Makimura, K. (Teikyo Univ.)	Microbe-1: Conducted in 2009	(3, 9, 11)
Microbe-2	International Space Station and their relationship to health problems (combined with “Microbial monitoring on the ISS”) (selected in 2007)		Microbe-2: Conducted in 2011	
Microbe-3		Nasu, M. (Osaka Univ.)	Microbe-3: Conducted in 2012	

Microbe-A	On-board microorganism monitoring in spacecrafts (selected in 2010)	Yamazaki, T. (Teikyo Univ./JAXA)	Experiment definition ongoing	http://iss.jaxa.jp/en/kiboexp/theme/second/pmlatter/yamazaki_e.pdf

Microbe-4	“Microbe”: Microbial monitoring in the International Space Station—KIBO (selected in 2012)	Nasu, M. (Osaka Univ.)	Experiment definition ongoing	http://iss.jaxa.jp/en/kiboexp/scenario/selection/fy2014_general/
